# Prodrug Strategies for Paclitaxel

**DOI:** 10.3390/ijms17050796

**Published:** 2016-05-23

**Authors:** Ziyuan Meng, Quanxia Lv, Jun Lu, Houzong Yao, Xiaoqing Lv, Feng Jiang, Aiping Lu, Ge Zhang

**Affiliations:** 1 Institution for Advancing Translational Medicine in Bone & Joint Diseases, School of Chinese Medicine, Hong Kong Baptist University, Hong Kong 999077, China; 15484572@life.hkbu.edu.hk (Z.M.); lvquanxia@163.com (Q.L.); ljaaa111@163.com (J.L.); yaohouzong@163.com (H.Y.); 2 Research Group of Precision Medicine and Innovative Drug, HKBU (Hong Kong Baptist University) (Haimen) Institute of Science and Technology, Haimen 226100, China; lxqd1@126.com; 3 The State Key Laboratory Base of Novel Functional Materials and Preparation Science, Faculty of Materials Science and Chemical Engineering, Ningbo University, Ningbo 315211, China

**Keywords:** paclitaxel, poor water solubility, low selectivity, prodrug

## Abstract

Paclitaxel is an anti-tumor agent with remarkable anti-tumor activity and wide clinical uses. However, it is also faced with various challenges especially for its poor water solubility and low selectivity for the target. To overcome these disadvantages of paclitaxel, approaches using small molecule modifications and macromolecule modifications have been developed by many research groups from all over the world. In this review, we discuss the different strategies especially prodrug strategies that are currently used to make paclitaxel more effective.

## 1. Introduction

**Figure 1:**
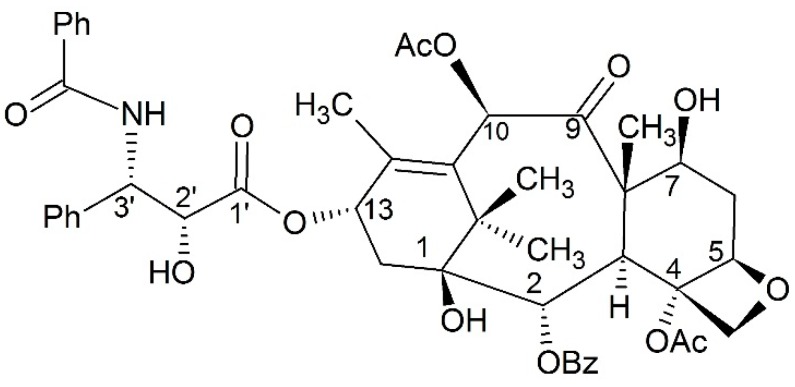
The structure of paclitaxel.

Paclitaxel (Figure 1) was isolated from *Taxus brevifolia* and its anticancer activity first reported in 1971 [1]. Additionally, it was approved as a kind of microtubule stabilizing agent in 1992. As an anticancer drug, paclitaxel can promote tubulin polymerization and stabilize microtubules from depolymerizing [2]. These procedures are relevant to special regions on tubulin including H6–H7 and the M-loop [3]. Paclitaxel is also able to interact with tubulin assemblages at low temperatures, as well as without GTP or microtubule-associated proteins [4]. The binding affinity of paclitaxel and tubulin depends on the nucleotide content of tubulin [5]. Due to this unique anticancer mechanism, paclitaxel has aroused much interest for further development. Besides its anticancer activity, paclitaxel can exert a variety of positive influences on the immune system [6], and play a potential role in treating neurodegenerative diseases as well as inhibiting botulinum neurotoxin [7,8].

Although paclitaxel is effective for various human diseases, it is also faced with limitations: first, paclitaxel has extremely poor water solubility and it needs a relatively higher dose to take effect compared to other anticancer drugs. For these reasons, it is always administered with ethanol and Cremophor EL as vehicles to increase its water solubility, which may cause severe hypersensitivity in patients. Hence, to avoid this hypersensitivity and obtain better clinical use of paclitaxel, developing a new co-solvent [9,10,11] and improving the formulation for paclitaxel delivery systems has become important [12]. In recent years, outstanding drug delivery systems for paclitaxel have been developed, and different carriers have been employed, such as cubosomes [13], β-cyclodextrins [14,15], gold microplate [16], gold nanorod [17], lipid vesicles [18], microparticles [19], nanoparticles [20,21], micelles [22,23,24,25,26], and liposomes [27].

Other than its low water solubility, paclitaxel is also faced with multiple drug resistance like other anti-cancer agents. Specific excipients or administered in combination with other drugs were used to solve this limitation [28]. Also many formulations have been under clinical trials or investigation [29]. One research showed that better efficacy of paclitaxel could be obtained by prescribing with carboplatin which could produce a synergistic effect [30]. In the same strain, with the development of anticancer agents for paclitaxel resistant cancer cells, new chances for paclitaxel’s combination have been provided [31]. In recent years, paclitaxel has also been delivered with microRNA, shRNA and siRNA to get better therapeutic effects [32,33,34]. Furthermore, it also shows dose limiting side effects, which are common to other cytotoxic agents [35].

In addition to developing better formulation for paclitaxel, there were mainly two ways to achieve more efficient modified paclitaxel. One was to synthesize various paclitaxel analogs. These strategies included hybridizing paclitaxel with other chemicals, blocking paclitaxel’s metabolic sites, locking the binding conformation by bridging converts or directly modifying its structure [36,37,38,39,40]. These paclitaxel analogs were more efficient with regard to water solubility and anticancer activity [41]. The other way was to transfer paclitaxel into a prodrug which can release free paclitaxel after administration. Through this way, the shortcomings of paclitaxel as described above could be largely overcome. These prodrugs included small molecule prodrugs and macro molecule prodrugs. Most of the small molecule prodrugs were designed to overcome paclitaxel’s low water solubility. For the macro molecule prodrugs, paclitaxel was conjugated to polymers or protein. This strategy could commonly increase the targeting ability of paclitaxel by enhanced permeability and retention effects (EPR) as for other macro molecule prodrugs [42]. Herein, various prodrug strategies that have been currently used to make paclitaxel more effective are presented.

## 2. Structure and Activity Relationships

As for prodrug strategy, understanding the structure and activity relationship is the first step. Pioneer work showed that the central part of paclitaxel is rigid to a change in structure, while its side chain tail can be flexible [43]. In the central part of paclitaxel, the 1-OH and 2-benzoyloxy are important to paclitaxel’s anti-cancer activity [44]. Additionally in the flexible side chain, the activity of paclitaxel can be influenced by the stereochemistry of C2′ and C3′ [45]. Furthermore, another work postulated C2′–OH to be the bonding site of paclitaxel to tubulin [46]. Therefore, it was proposed to be the most important functional group in the region of the C-13 side chain which is a basic component for paclitaxel [47,48]. Thus, early development mainly focused on the C2′-position and C3′-position. Although C-7 to C-10 of paclitaxel does not interact with tubulin directly, it had been postulated that changes of this region might affect its bonding affinity to P-glycoprotein which is responsible for the MDR phenotype [49].

Due to its complicated structure, paclitaxel can be modified at different positions to obtain paclitaxel analogs, such as 2′-OH [50,51], 3′-position [49,52], C-2 position [53], C-4 position [54,55,56], C-7 position [49], C-10 position [57], C-13 position [58] or d-ring [59]. The macrocyclic paclitaxel analog had also been synthesized according to the structure activity relationship [60]. However, for prodrug strategy, C2′–OH has been the most commonly adopted, as it may be the binding site of paclitaxel to tubulin. At the same time C7–OH has also been adopted by some other groups.

## 3. Small Molecule Paclitaxel Prodrug

In this part, approaches to build up of small molecule paclitaxel prodrugs are introduced, including non-targeting prodrugs and targeting prodrugs.

### 3.1. Non-Targeting Modification

#### 3.1.1. Hydrophilic Modification

As paclitaxel’s anticancer mechanism became unfolded, making it more water soluble or bio-active was crucial before its clinical use. Two opposite approaches were adopted by different groups. One was to make paclitaxel prodrug more water soluble directly in order to get better bio-compatibility. The other way was to make paclitaxel more hydrophobic which was suitable for long half-life hydrophobic formulation. The hydrophobic modification will be introduced in the next section. Both ways were feasible for developing paclitaxel prodrugs.

##### Skeletal Migration

**Figure 2:**
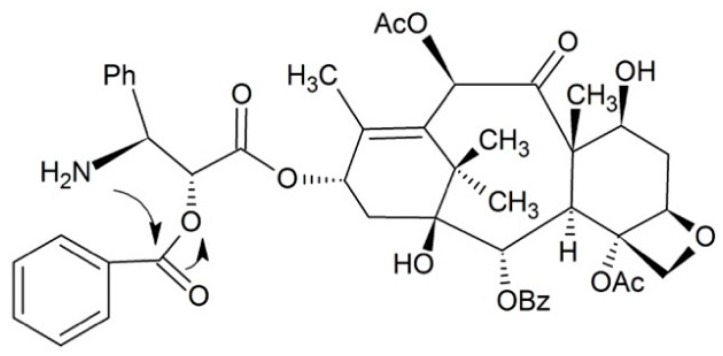
Skeletal migration approach for paclitaxel prodrug.

Hayashi and co-workers developed the ammonium salt of isopaclitaxel with good water solubility (Figure 2). After administrating, the isopaclitaxel could pH dependently form paclitaxel via O–N acyl migration without auxiliary and byproduct. The isopaclitaxel has great advantages with respect to toxicology and medical economics [61]. However, the results of *in vivo* study are still missing, thus experiments need to be performed to confirm its efficacy.

##### Phosphate Esters

**Figure 3:**
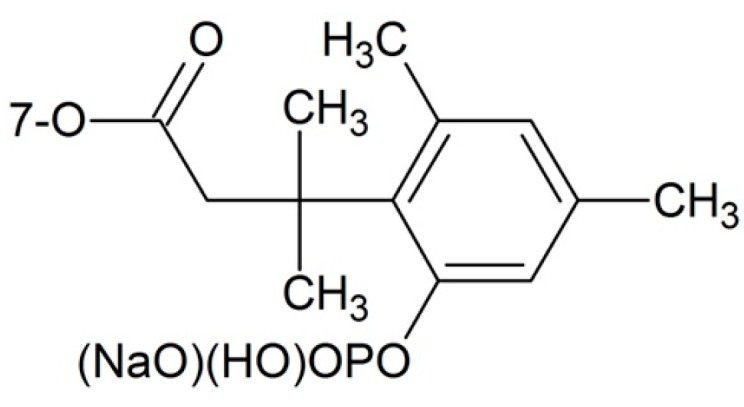
Trimethyl lock strategy.

In drug design, forming phosphate esters is a common strategy making the drug more soluble in water. In addition to good water solubility, the phosphate ester can also be a site of the substrate for alkaline phosphatases, this property enables the paclitaxel prodrug to have a fast release of the parent drug. Some simple phosphonooxymethyl ethers of paclitaxel were synthesized by introducing phosphate moieties to 2′-OH and 7-OH. The resulting prodrug had greatly improved water solubility and could release the free paclitaxel upon incubation with plasma and alkaline phosphatase [62]. In order to release free paclitaxel easier, Ueda and co-workers [63] introduced phosphate groups to 7-OH of 2′-ethoxycarbonypaclitaxel. In their design, the “trimethyl lock” was used to accelerate the release of the parent drug as a substrate of alkaline phosphatases (Figure 3). The water solubility is 2.5–5 mg/mL, and some of the compounds exhibit comparable *in vivo* cytotoxicity in the M109 murine tumor model.

##### Others

**Figure 4:**
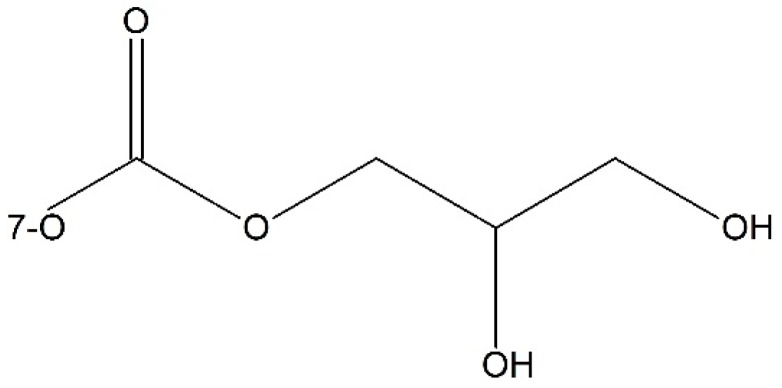
The pH-dependent cleavable dihydroxypropyl side chain.

Besides phosphate esters, there were other methods to improve the water solubility of paclitaxel. Such as, Damen *et al.* [64] synthesized two paclitaxel esters of malic acid at 2′-OH and 7-OH respectively. The resulting prodrugs were stable in plasma, and exhibited improved water solubility, antitumor activity, and less cytoxicity. Niethammer *et al.* [65] found when 7-OH was blocked by a pH-dependent cleavable dihydroxypropyl side chain, the afforded prodrug could achieve equal anti-tumor activity but was 50-fold more water soluble compared to free paclitaxel (Figure 4).

#### 3.1.2. Hydrophobic Modification

The other way for the small molecule non-targeting paclitaxel prodrug was to make paclitaxel more hydrophobic by linking a hydrophobic moiety at the 2′-OH position. These hydrophobic paclitaxel prodrugs were more suitable for a nanoparticle delivery system with a longer half life in circulation [66].

##### Silicate Esters

In order to achieve a wider water solubility range of the paclitaxel prodrug, 2′-OH was silicated with different alkyl groups. By changing the alkyl group, the water solubility could shift to a proper range for formulation [67]. Han and co-workers synthesized silicate derivatives of paclitaxel as prodrugs. In their study, the alkyl group in the silicate derivatives was chosen to generate prodrugs with greater hydrophobicity for preparing nanoparticles, and then a formulation with maximum loading capacity of 75 wt % of prodrug and greater *in vitro* efficacy was prescribed. Table 1 shows cLogP and t_1/2_ of silicated paclitaxel prodrugs with different alkyl groups [68].

##### Squalenoylation Technology

Other than silicate esters, squalenoylation technology has also been developed for delivering poorly water soluble therapeutic agents. When a hydrophilic polyethylene glycol (PEG) linker is introduced between hydrophobic paclitaxel and squalene, the water solubility can be adjusted by selecting the most suitable length of the PEG linker and squalene moiety. A series of paclitaxel prodrugs was synthesized by Dosio and co-workers [69]. All the prodrugs can self-assemble into nanoparticles in low concentration and are stable in water for several weeks. Preliminary biological studies showed these squalenoyl-paclitaxel nanoassemblies can induce the HT-29 and KB-31 cells’ microtubule bundles from forming, and it also exhibited notable antitumor activity on a lung tumor cell line. Overall this technology has potential for delivering a poorly soluble drug.

#### 3.1.3. Mutual Drugs

**Figure 5:**
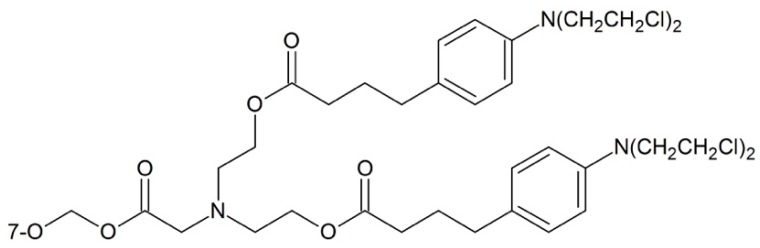
Chlorambucil-paclitaxel prodrug.

Mutual prodrug strategy can contribute a lot to obtain paclitaxel prodrugs with higher anti-cancer efficiency and increased water solubility. Wittman *et al.* [70] reported that the combination of paclitaxel with other antitumor agents could afford improved cytotoxicity to MDR cell lines. In the study, a series of chlorambucil–paclitaxel prodrugs were synthesized, and the compound exhibited in Figure 5 was demonstrated to have vigorous antitumor activity *in vivo*, based on M109 murine models and paclitaxel resistant M109/taxlR models.

**Figure 6:**
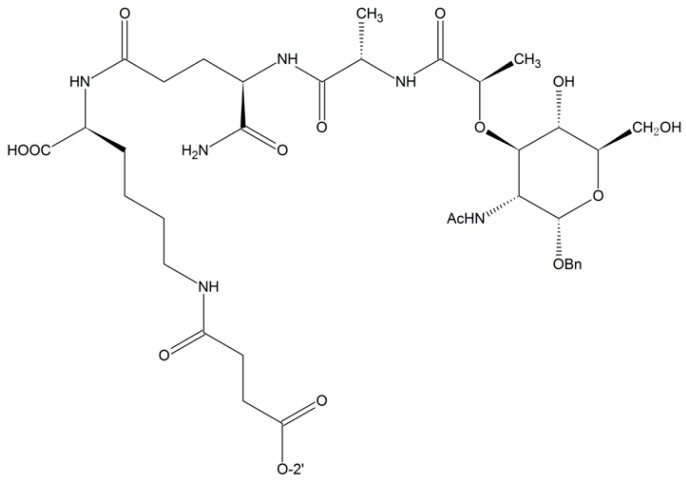
Muramyl dipeptide-paclitaxel prodrug.

Muramyl dipeptide (MDP) which can elicit human immunological responses is the minimal structure of Gram-positive and the Gram-negative bacteria’s cell walls. Paclitaxel prodrugs containing MDP or its analog motifs can elicit human immunological responses, and thus achieve a synergistic anticancer effect. This strategy can combine immunotherapy and chemotherapy together in curing cancer. The dipeptide-paclitaxel prodrugs in which the dipeptide was conjugated to the 3′-amino group, 2′- and 7-hydroxyl group of the paclitaxel were synthesized respectively (Figure 6). Among them, the 2′-OH prodrug was around 200 times more water-soluble than paclitaxel [71]. However, further researches showed that it was not powerful in antitumor activity. Thus, further chemical modification of this prodrug was conducted by the group for better efficacy [72].

Caron and co-workers linked paclitaxel and gemcitabine together via a short polyisoprenoyl spacer. This series of bolaform polyisoprenoyl paclitaxel and gemcitabine prodrug can form nanoassemblies with a diameter of 100–200 nm due to the property of the spacer. These nanodevices with high drug loading rate showed improved *in vitro* activities on several human and murine cancer cell lines compared to those nanoassemblies of the squalenoyl drugs solely or in combination. Thus, this preparation method is potent for nanoparticle mediated combination therapy [73].

**Figure 7:**
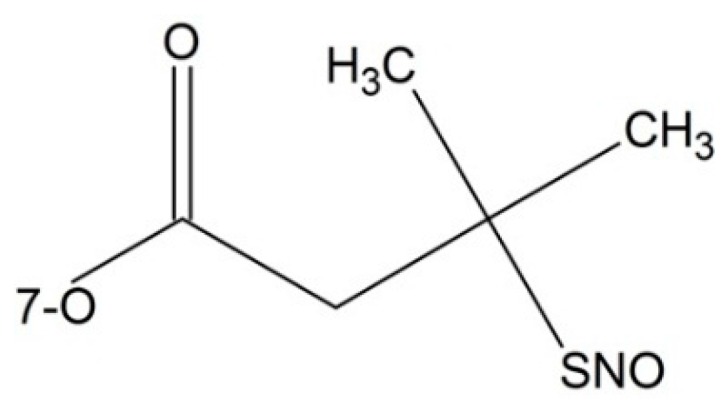
Paclitaxel prodrug containing nitric oxide donor.

Besides enhancing antitumor activity, the antistenotic profile of paclitaxel can also be increased via mutual drug strategy. An early attempt was conducted by Vrudhula and co-workers [74]. They synthesized the captopril-paclitaxel mutual prodrug, and the antistenotic profile was obviously increased. Recently paclitaxel was conjugated to polyisobutylene for controlled release from vascular stent coating, the results showed that this coated stent was potent for clinical use [75]. In order to achieve better therapeutic effects of paclitaxel coated stents, adamantine nitrosothiol was introduced at the 7-position as a nitric oxide donor (Figure 7). Stents coated with this modified paclitaxel were 34% better than paclitaxel coated stents, and 41% better than polymer coated stents [76].

#### 3.1.4. Dipeptide Prodrugs

As mentioned above, paclitaxel shows dose limiting side effects [35]. In order to reduce such side effects, Dubowchik *et al.* [77] conjugated a cathepsin-B sensitive dipeptide (Phe–Lys) to both the 2′-OH and 7-OH of the paclitaxel and a self-immolative PABC (*p*-aminobenzyloxycarbonyl) linker was employed to avoid steric interference between the dipeptide and paclitaxel. They found the resulting prodrugs were stable in plasma and could release the paclitaxel when internalized by tumor cells. In addition, the prodrug in which the modified group was the 7-OH showed a longer half-life of 66 min in rat liver lysosome than the 2′-OH of 19 min. Although the 7-OH showed a shorter half-life of 40 min in cathepsin B solution, the 2′-OH modification was 9 h.

#### 3.1.5. Others

Paclitaxel could bind not only to tubulin but also to P-glycoprotein [78]. A paclitaxel prodrug with succinate at the C10 position showed low affinity to P-glycoprotein which could enable the paclitaxel analog to pass through the blood-brain-barrier. In this way, the paclitaxel concentration is enhanced in the brain about 3-fold. Meanwhile cytotoxic research showed the prodrug could retain comparable efficacy towards the breast cancer line MCF7 with an IC_50_ of 35.7 nm compared to paclitaxel’s IC_50_ on this cell line which is 1.8 nm [79].

**Figure 8:**
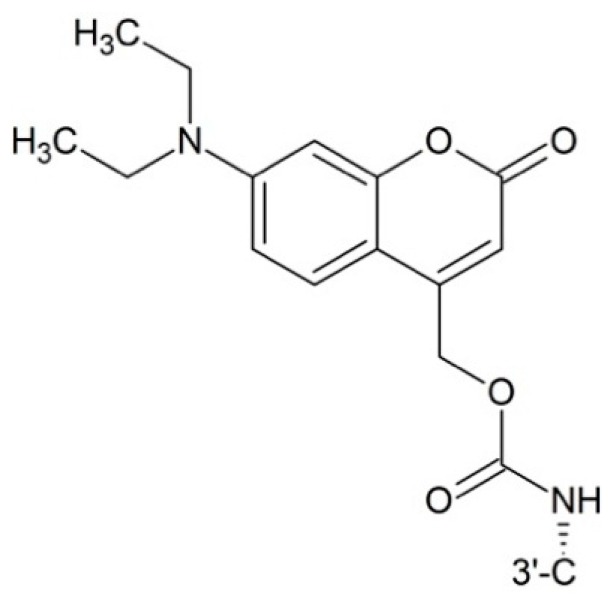
Photo responsive paclitaxel.

Photodynamic therapy (PDT) is a type of method for treating cancer. After a sensitizer is administered, the pathological area is exposed to visible light. The beam of light can activate a sensitizer to release a cytotoxic free radical or singlet oxygen. Skwarczynski and co-workers [80] first synthesized photo responsive paclitaxel by introducing 7-*N*,*N*-diethylamino-4-hydroxymethyl coumarin (DECM) as a photolabile group to 2′-benzoyl-paclitaxel which could increase paclitaxel’s water solubility by transforming it into chloride and be activated at 430.6 nm without decomposition (Figure 8). After activation by light, the cleavage of the carbamate induces O–N acyl migration to end up with paclitaxel. Noguchi and co-workers [81] also synthesized a coumarin-based high water soluble paclitaxel prodrug, which could release the parent drug at 365 nm UV light.

Besides the above, the DHA-Paclitaxel prodrug is a very important compound. This prodrug strategy is realized by the conjugate DHA molecule to 2′-OH and is tested in the M109 mouse tumor model. Results show tumor AUCs for DHA-paclitaxel are 61-fold higher at equitoxic doses and 8-fold higher at equimolar doses than palitaxel [82].

### 3.2. Targeting Modification

#### 3.2.1. Targeting Tumor Overexpressed Enzymes

Tumor tissues are different from normal tissues in various aspects. One difference is that some enzymes are over expressed in tumor tissues. By employing a tumor overexpressed enzymes recognition motif paclitaxel prodrugs can show selective cytotoxicity towards tumor tissues.

##### Targeting β-d-Glucuronidase

**Figure 9:**
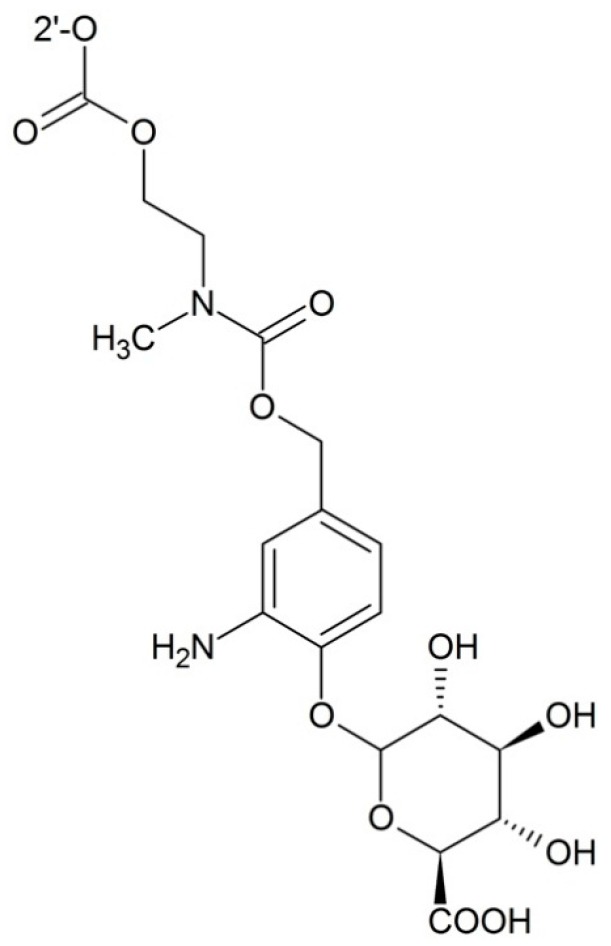
Targeting β-d-glucuronidase prodrug.

β-d-Glucuronidase is a kind of extracellular enzyme in necrotic tumors. Prodrugs in which the paclitaxel is modified with β-glucuronide can increase the selectivity and the water solubility of paclitaxel. By attaching β-glucuronide and a self-immolative spacer to the 2′-position of the paclitaxel, Alaoui *et al.* [83] successfully developed a strategy to obtain a plasma stable and enzyme cleavable prodrug which could be used for prodrug monotherapy (PMT) and antibody directed enzyme prodrug therapy (ADEPT) (Figure 9). The IC_50_ of this compound is 11.3 nm compared to free paclitaxel 0.16 nm on HT-29, but further *in vivo* study needs to be carried out.

##### Targeting Prostate-Specific Antigen (PSA)

**Figure 10:**
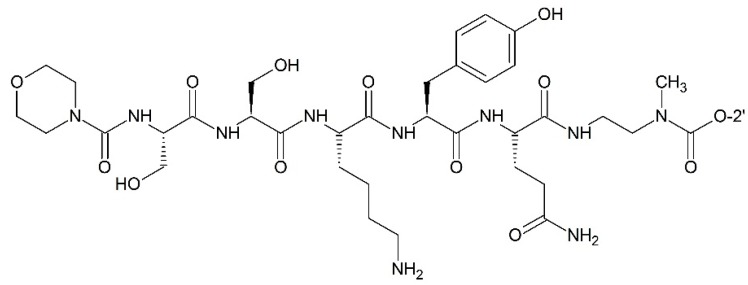
Targeting PSA (prostate-specific antigen) paclitaxel prodrug.

Different peptide moieties can be recognized and decomposed by the different corresponding enzymes. When attached to peptide moieties, paclitaxel can obtain target ability. Elsadek and co-workers [84] designed and prepared EMC–Arg–Ser–Ser–Tyr–Tyr–Ser–Leu–PABC–paclitaxel (EMC: ε-maleimidocaproyl; PABC: *p*-aminobenzyloxycarbonyl) which contained a prostate-specific antigen (PSA) cleavable peptide site. EMC in the compound was an active moiety which enabled the conjugate to bind to albumin, and be retained by the vessels in the prostate. These properties made the conjugate promising for future development in curing prostate cancer. Kumar and co-workers [85] also synthesized paclitaxel prodrug targeting PSA by taking advantages of HSSKLQ (His–Ser–Ser–Lys–Leu–Gln) or SSKYQ (Ser–Ser–Lys–Tyr–Gln) peptides which could be cleaved by PSA. The peptides and the paclitaxel were linked with para-aminobenzyl alcohol (PABS) or ethylene diamine (EDA). Inducing these linkers resulted in an increased hydrolysis rate of the prodrug by PSA. The anti-cancer activities of these prodrugs were conducted on various cell lines, including CWR22Rv1 prostate cancer cell line. Among these prodrugs, the compound in which the peptide and the paclitaxel were linked with ethylene diamine was stable and could be efficiently converted into free paclitaxel that kills cancer cells in the presence of PSA (Figure 10).

Besides targeting β-d-glucuronidase and targeting PSA paclitaxel prodrugs, targeting the plasmin prodrug of paclitaxel has also potential for future use [86].

#### 3.2.2. Targeting Transporters

**Figure 11:**
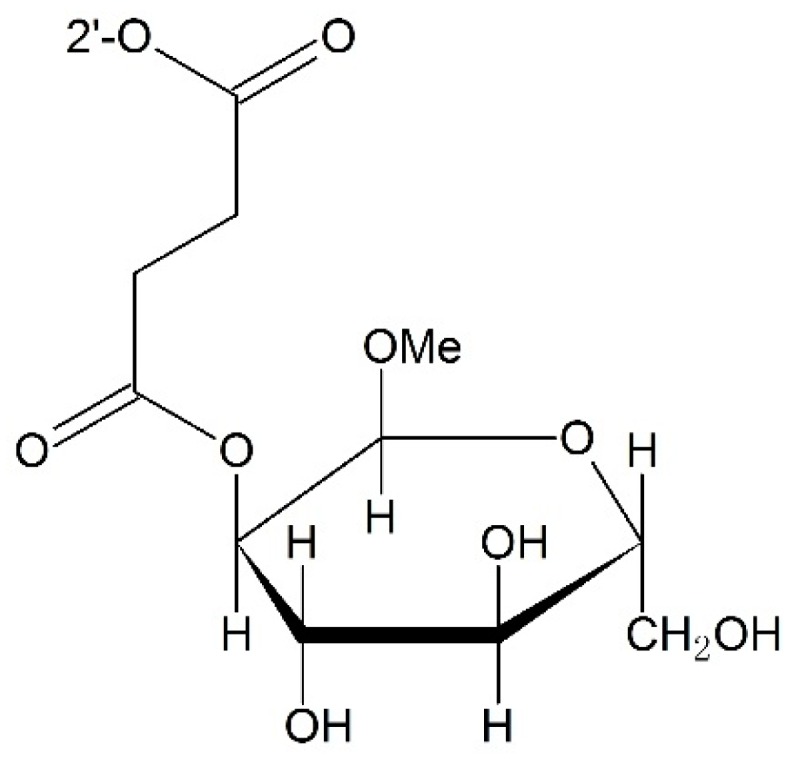
Targeting GLUTs (facilitative glucose transporters) prodrug.

Facilitative glucose transporters (GLUTs) are a kinds of transporters which are responsible for uptaking glucose into cells. By attaching 2′-glucopyranose to the 2′-OH of the paclitaxel via succinic acid, Liu *et al.* [87] obtained a good paclitaxel analogue with high selectivity towards GLUTs. To further improve the water solubility of paclitaxel, Lin and co-workers [88] prepared a series of 2′-paclitaxel conjugates by employing the 2′-glucose or glucuronic acid motif. These prodrugs showed enhanced water solubility and great selectivity towards GLUTs which were overexpressed in tumor cells (Figure 11).

#### 3.2.3. Targeting Hypoxia

**Figure 12:**
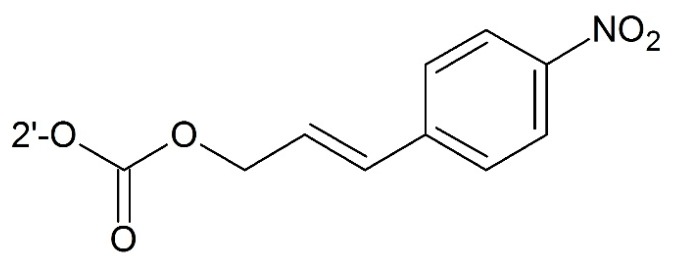
Targeting hypoxia prodrug.

Hypoxia is a phenomenon of low oxygen concentration in tissues. In solid tumor, mild oxygen deficiency (hypoxia) or severe oxygen deficiency (anoxia) regions can be usually observed. In these parts, due to inefficient blood supply, drugs cannot reach their targets easily. In addition, hypoxic cells may also be resistant to both radiotherapy and conventional chemotherapy [89]. Hence, developing bioreductive (pro)drugs targeting hypoxia with low side effects is necessary. Damen *et al.* [35] first prepared a bioreductive paclitaxel prodrug by attaching the aromatic nitro and azido groups bioreductive trigger to C2′–OH. For blocking C2′–OH, lower side effects could be achieved. When reaching hypoxia regions, free paclitaxel was released after reduction of the nitro group and 1,6-elimination of a 4-amino or 4-hydroxylamino benzyloxycarbonyl moiety (Figure 12).

#### 3.2.4. Targeting Integrin

**Figure 13:**
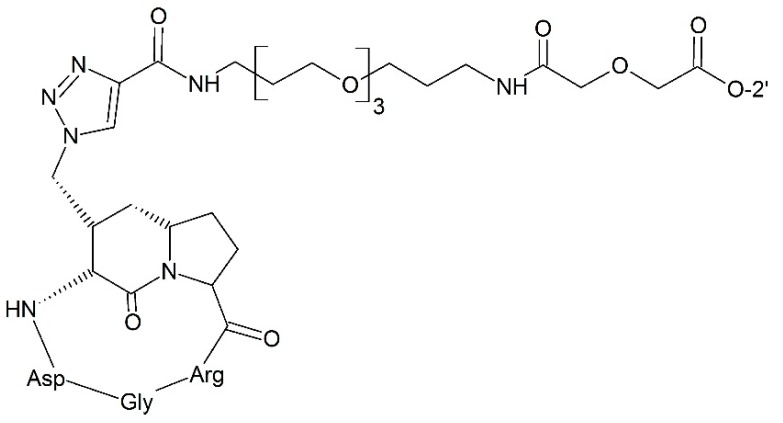
Targeting integrin prodrug.

Integrin is an important protein in promoting cell attachment and migration for tumor cells. Pilkinton and co-workers [90] synthesized a series of conjugates by linking paclitaxel to either cyclic AbaRGD (Azabicycloalkane-RGD) or AmproRGD (Aminoproline-RGD) integrin recognizing matrices with various linkers. The results showed these conjugates had satisfactory binding affinity towards the integrin, excellent cell sensitivity, and remarkable antitumor activity (Figure 13). Zhang *et al.* [91] also conjugated paclitaxel to *cyclo*-(Arg–Gly–Asp-d-Phe–Lys) (c[RGDfK]) for treating glioma tumor. In addition, the cyclo [DKP-RGD] peptidomimetics-paclitaxel conjugate and dimeric RGD peptide-paclitaxel conjugate also achieved a superior antitumor effect against the IGROV-1/Pt1 human ovarian carcinoma xenotransplanted in nude mice compared to paclitaxel [92,93].

#### 3.2.5. Targeting Receptors

Folic receptor-α (FA-α) expresses little in normal cells and even not be detectable. Nevertheless, it is upregulated and can be detected in some kinds of cancer cells. Folic acid is the ligand of FA-α and therefore can target drugs to such kind of cancer cells with high expression of FA-α. Shan *et al.* [94] synthesized multi-small molecule conjugations in which folic acid was conjugated to paclitaxel at the 2′-position via single amino acids (Arg or Glu). This resulting prodrug showed improved water solubility. Besides, this prodrug showed increased uptake by FR-α over expressing tumor cells (*i.e.*, MCF-7, MDA-MB-231, and A549) when compared to normal HEK293 cells. This suggests the prodrugs have good anticancer activity and targeting ability.

Taking advantages of receptor bonding peptide is an efficient way for targeting delivery, Ndungu *et al.* [95] synthesized the conjugate in which the tissue factor binding peptide fVIIa was attached to paclitaxel at the C2′ or C7 position via a succinic acid linker. The conjugate showed better anti-cancer activity towards human head and neck squamous KB3-1 cells.

#### 3.2.6. Targeting Glutathione

**Figure 14:**
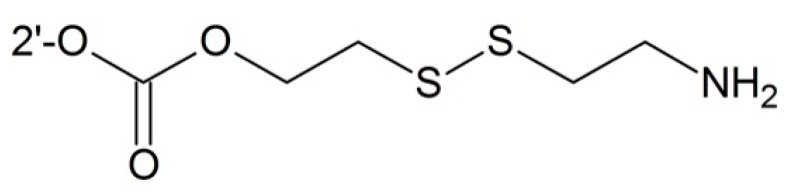
Targeting glutathione prodrug.

Many tumor cells contain a high concentration of glutathione which can be used to targeting delivery of the drug to tumor cells. Taking advantages of intracellular sulfhydryl-containing spices such as glutathione (GSH) or its thiolate anion GS^−^ at a biological pH attacking the disulfide bond, Gund *et al.* [96] designed various prodrugs containing disulfide linker which showed better water solubility and anticancer activity than free paclitaxel (Figure 14).

#### 3.2.7. Prodrugs for Antibody Directed Enzyme Prodrug Therapy (ADEPT)

**Figure 15:**
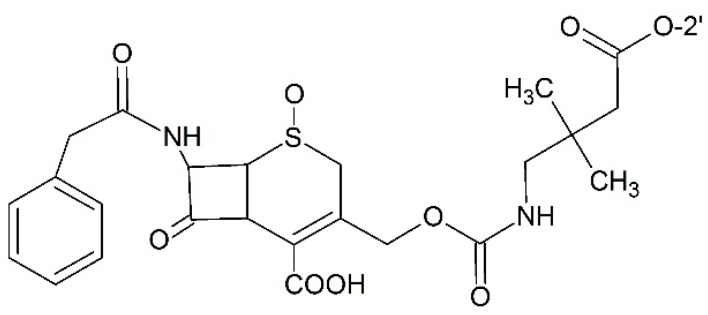
Prodrug for antibody directed enzyme prodrug therapy (ADEPT).

ADEPT is the strategy aiming to achieve selective toxicity to tumor cells meanwhile keeping normal cells undamaged from stand by effects. The monoclonal antibody enzyme fusion protein is administered before cytotoxic prodrug in this strategy. For high affinity of monoclonal antibody, monoclonal antibody enzyme fusion protein can concentrate in tumor tissues. The subsequent prodrug is activated by the enzyme part in the antibody enzyme fusion protein and thus cytotoxicity appears. Using this strategy, Vrudhula and co-workers [97] synthesized the cephalosporin prodrug of paclitaxel with self-immolative linkers which could be activated by l-49-sFv-β-Lactamase fusion protein (Figure 15). Another example of prodrug for ADEPT was designed by Bont *et al.* [98]. In their study, paclitaxel was connected to β-glucuronic acid via a carbamate linkage which could release the free drug under the presence of β-glucuronidase which demonstrated desirable anti-tumor efficacy. Also, this design is potent for ADEPT.

#### 3.2.8. Prodrugs for Gene-Directed Enzyme Prodrug Therapy (GDEPT)

Ishida and co-workers [99] designed a paclitaxel-2′-ethylcarbonate prodrug, this prodrug converted into free paclitaxel with the help of an HSV amplicon expressing rabbit-carboxylesterase (CES) with HF10, an attenuated replication-competent virus, as a helper virus which could be used in cancer virotherapy. This approach can produce CES at the tumor sites with a high level, thus free paclitaxel can be released from the prodrug, and enhance the efficacy of HF10.

## 4. Macromolecular Prodrugs

As mentioned above, formulation of paclitaxel is of vital importance in order to avoid complicated formulations. Macromolecular paclitaxel strategies have been developed by some research groups. Macromolecular paclitaxel can not only avoid complicated formulations but also enhance the targeting ability of paclitaxel by improved permeability and retention effects (EPR). In this section, several representative macromolecules which were used to prepare the paclitaxel prodrug are discussed.

### 4.1. Polyethylene Glycol (PEG)

#### 4.1.1. PEG as Drug Carriers

Initially, PEG was used as a dissolution aid for paclitaxel [100]. In order to get a more stable prodrug, a series of PEG-paclitaxel conjugates were prepared by different groups. When paclitaxel was conjugated to PEG via an amino acid spacer, the water solubility would be remarkably enhanced [101]. Greenwald *et al.* [102] prepared the conjugate in which the paclitaxel was attached to ~40 kDa PEG via an ester bond. The water soluble conjugate was shown to be relatively nontoxic compared to paclitaxel. However, increased toxicity was observed in the living expectancy in P388-treated mouse. Liang and co-workers [103] synthesized PEG paclitaxel conjugate via a cathepsin B cleavable linker (valine-citrulline) and a PABC spacer. The afforded conjugate showed significant advantages in terms of high water solubility, without toxic excipients, and tumor environment sensitive drug release.

#### 4.1.2. PEG Copolymer Prodrugs

Although PEG is potent for paclitaxel conjugates, it is also challenged by its intrinsic property that it has only two drug loading sites at each end of the polymer. This limitation prompted the development of PEG copolymers.

Gu and co-workers [104] established a versatile platform for using prodrug micellar nanoparticles to deliver paclitaxel. Unlike other noncovalently bonding nanosystems, paclitaxel was acetal-linked to water soluble poly(ethylene glycol)-*b*-poly(acrylic acid) (PEG–PAA) block copolymer. The prodrug was pH dependent degradable and thus, paclitaxel could be released rapidly. These paclitaxel prodrug nanoparticles showed high antitumor activity to KB and HeLa cells (IC_50_ = 0.18 and 0.9 μg PTX equivalent (equiv.)/mL, respectively) as well as A549 cells, a PTX-resistant. The system showed superior anticancer activity to both drug sensitive and resistant cancer cells with remarkable efficient drug content (up to 42.8 wt % paclitaxel). Using this strategy, the drug loading ability of the polymer could be improved by a large amount.

Besides the drug loading ability, the co-polymer is more functional than the original PEG. Yu *et al.* [105] synthesized a functional polylactide-*g*-paclitaxel-poly(ethylene glycol) drug conjugate. In this conjugate, paclitaxel was used as a divalent agent bridging degradable azide-functionalized polylactide (PLA)-based backbone and PEG side chain. *In vitro* study showed the azide motif could be hydrolyzed faster at pH 5.5 than pH 7.4 while the ester bond is more stable at pH 5.5 which proved the conjugate was potent to release paclitaxel faster in tumor tissues.

Lv *et al.* [106] synthesized the 3,3′-dithiodipropionic acid functionalized poly(ethylene glycol)-*b*-poly(l-lysine) (mPEG-*b*-P(LL-DTPA)) and paclitaxel was conjugated to this polymer. Not surprisingly, it was a redox dependent drug release conjugate. Besides, the prodrug was pH dependent release and exhibited high cytotoxic towards tumor cells compared to non-sensitive micelles.

Furthermore, PEG copolymer can form self-assembly micelles, which can simplify the preparation process. Chen *et al.* [107] prepared co-polymers with PEG side chains, and paclitaxel was covalently conjugated to the polymer via disulfide linkers. These self-assembly micelles showed apparent cytotoxicity to OS-RC-2 kidney tumor cells and low cytotoxic to normal cells (macrophage cells).

Besides mono drug therapy, paclitaxel can also combine other drugs with the aid of PEG to formulate a drug delivery system. Zhu and co-workers [108] developed a nanopreparation which was composed of matrix metalloproteinase 2 (MMP2)-sensitive self-assembly PEG2000-paclitaxel conjugate, transactivating transcriptional activator peptide-PEG1000-phosphoethanolamine (PE) which could enhance cell-penetration, and PEG1000-PE as a nanocarrier building block. This system exhibited enhanced anticancer activity, and could deliver the drug into cancer cells.

#### 4.1.3. PEG Linker Prodrugs

In addition to employing PEG as a drug carrier directly or forming PEG co-polymers, PEG can also be used as a linker between carrier and paclitaxel. Bao and co-workers [109] synthesized d-α-tocopherol polyethylene glycol succinate-based paclitaxel prodrug which was self-assembly aggregate in micelles with high drug loading. The prodrug was designed by introducing P-glycoprotein (P-gp) inhibitor and a disulfide linker to realize redox-sensitive property in tumor tissues. The prodrug was not only 91% more efficient than paclitaxel but also had increased AUC and half-life.

The paclitaxel conjugates with PEG linker could achieve targeting ability by conjugating targeting moieties. Safavy and co-workers [110,111] linked paclitaxel-PEG conjugate with BBN peptide which could bind to the cell surface bombesin/gastrin-releasing peptide receptor. The resulting prodrug retained binding affinity as the original BBN, its IC_50_ was lower than free paclitaxel when tested on NCIH1299 human non-small dell lung cancer cell. In order to enhance the endocytosis of the prodrug, Yin and co-workers [112] synthesized octreotide-PEG-disulfide bond-paclitaxel conjugates. This design could realize not only targeting ability by EPR effects but also OCT-receptor mediated endocytosis. The results showed the design could be used for superior targeting redox-sensitive polymers.

### 4.2. Hyaluronic Acid (HA)

Hyaluronic acid has synergism effects with paclitaxel in inhibiting cancer migration [113]. Lee and co-workers [114] synthesized paclitaxel-hyaluronic acid conjugate via an ester bond. This conjugate could form self-assembly nanosized micellar aggregates in aqueous solution and exhibited more pronounced cytotoxicity for cancer cells overexpressing HA receptors. Yin *et al.* used cross linker containing disulfide bonds which is sensitive to glutathione to link paclitaxel and hyaluronic acid (HA) together. This system was also able to enhance the therapeutic efficacy of paclitaxel and provide a redox-responsive, controlled releasing, targeting platform for paclitaxel delivery [115].

### 4.3. N-(2-Hydroxypropyl)methacrylamide (HPMA)

*N*-(2-hydroxypropyl)methacrylamide (HPMA) is also a popular macromolecule used to prepare the paclitaxel prodrug. Miller and co-workers [116] conjugated alendronate and paclitaxel to HMPA via cathepsin B cleavable peptide in which alendronate was used to target bones. The resulting conjugate exhibited improved efficacy, and could be better tolerated and administered than when formulated in Cremophor and ethanol. Erez and co-workers [117] introduced cathepsin B cleavable linker and AB3 self-immolative space to form paclitaxel HPMA conjugates. The resulting conjugate exhibited higher drug loading and enhanced cytotoxicity on murine prostate adenocarcinoma cells when compared to a classic monomeric drug-polymer conjugate. In addition to peptide linker, other linker strategies were also developed. Etrych *et al.* [118] synthesized HMPA copolymer-paclitaxel conjugates by inducing hydrolytic cleavable linkage which was formed by the reaction of the hydrazide group-terminated side chain of the polymer with the carbonyl group of a drug derivative. *In vitro* study showed this conjugate could release paclitaxel faster at pH 5 compared to pH 7. Furthermore, this conjugate showed better anti-tumor activity in the 4T1 model of mammary carcinoma than the parent paclitaxel.

### 4.4. Dendrimers

Dendrimer is a kind of highly branched polymer. Although dendrimers are less popular than other polymers in delivering paclitaxel, a dendrimer delivery system has some advantages over other polymer delivery systems. It can form a monodispersed drug delivery system. Reproducible pharmacokinetics and pharmacodynamics could be observed from batch to batch. Some specific dendrimer paclitaxel carrier could also influence tubulin stability including polyamidoamine (PAMAM) [119].

**Figure 16:**
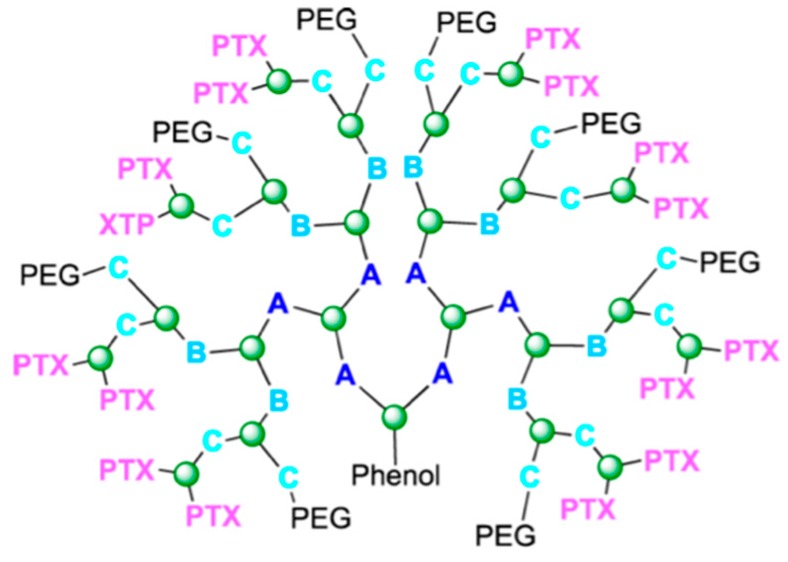
Dendrimer with 16 paclitaxel binding site and 8 PEG binding sites. Reproduced with permission from Reference [123]. Green atoms are 1,3,5-Trichlorotriazine, PEG is polyethylene glycol, PTX is Paclitaxel, A is 1,3-Bis(4-piperidyl)propane, B is Piperazine, C is 4-Piperidinemethanamine.

PEGylated triazine dendrimers containing 12 paclitaxel binding sites were synthesized with different ester or disulfide linkers between the core and the 2′-OH in paclitaxel [120]. This group also carried out *in vivo* experiments of these polymers. Just as they hypothesized, introducing a labile disulfide linker formed a more cytotoxic prodrug [121]. After this, PEGylated triazine dendrimers for paclitaxel delivery received further development [122]. Dendrimers of this kind with approximately 16 paclitaxel binding sites and eight PEG group (Figure 16) binding sites were built up in different ways. The best construction is shown in Figure 16 [123]. Apart from this, highly branched dendrimer can also protect siRNA from RNase digestion which makes dendrimer an ideal carrier for combining paclitaxel to siRNA. Kala and coworkers [124] reported that co-delivering paclitaxel and Akt siRNA by a triethanolamine-core poly(amidoamine) dendrimer showed great potential for treating ovarian cancer.

Satsangi and co-workers [125] synthesized paclitaxel poly(amidoamine) dendrimer conjugates with capthesin B cleavable peptide linker (Gly–Phe–Leu–Gly). This design enabled the conjugates to release paclitaxel in tumor tissues where capthesin B activity is relatively high compared to normal tissues. In this way the conjugates achieved markedly higher tumor reduction compared to paclitaxel in the MDA-MB-231 model.

For PEG drug conjugates, their intrinsic limitation is owing to PEG’s chemical structure where only the end can form conjugates. In order to get a higher amount of payload, Clementi *et al.* [126] developed a strategy giving PEG a dendrimer at each end. By using this method, paclitaxel and alendronate were conjugated to the H_2_N–PEG–dendrimer–(COOH)_4_, this conjugate exhibited an increased half-life, and could be resolved in the physical environment without Cremophor EL.

### 4.5. Other Polymers

In order to avoid complicated formulation, paclitaxel was conjugated to low molecular weight chitosan with a cleavable ester bond. As a result, water solubility was enhanced to >1 mg/mL with comparable IC_50_, and the prodrug could be administered orally [127].

There is another property of paclitaxel which has directed the development of macromolecular paclitaxel prodrugs. It had been shown that paclitaxel’s efficacy is more dependent on exposure time rather than on its concentration [128]. Therefore, a slow release system is more efficient in delivering paclitaxel. Cavallaro *et al.* [129] first used α,β-Poly(*N*-2-hydroxyethyl)-dl-aspartamide (PHEA) whose properties were similar to plasma as paclitaxel succinic anhydride derivative carriers linked by an ester bond whichcould be cleaved by an enzyme. This system showed high drug loading, stable in plasma, and prolonged drug release. By using polylactide, Yu and co-workers [130] designed brush polymer for azide-functionalized paclitaxel sustained delivering.

Delela [131] synthesized the conjugate in which paclitaxel was attached to poly (styrene-co-maleic acid), this self-assembly formed nanoparticles exhibiting pH dependent release, increasing in *C*_max_ and half-life, and could be detained in the tumor via an enhanced permeability and retention effect (EPR).

Wang and co-workers [132] synthesized heparin-paclitaxel conjugates via a single amino acid linker, in which heparin could inhibit tumor development. Results showed the anticoagulant activity of the prodrug was decreased sharply compared to heparin. This meant the prodrug strategy was safe to be administered systemically. This prodrug did not only have a self-assembly property in preparation, but also exhibited better cell inhibition for MCF-7 cells than free paclitaxel. The targeting capacity to solid tumor was also enhanced in this design.

### 4.6. Proteins

Aiming to avoid using Cremophor EL, choosing proper drug formulation or carrier is vitally important. Dosio and co-workers [133] conjugated paclitaxel to human serum albumin. The afforded conjugate became biocompatible and could be internalized into cells followed by drug release inside the cell. This conjugate could also release parent drug continuously to provide a depot effect.

Conjugating to antibody is an efficient approach for getting better target ability. Using this method, drugs can be specifically delivered to tumor sites and fewer side effects could be observed. Safavy and co-workers [134] conjugated paclitaxel to anti-epidermal growth factor receptor (anti-EGFR) momoclonal antibody Erbitux (C225) via a succinic acid linker. With regard to the conjugate, the 24 h tumor uptake was not significantly different from the original Mab, which meant paclitaxel bonding did not affect the antigen-binding and original inhibiting properties of C225, an early cleavage of the drug was observed. After this, glutaric acid linker was employed for avoiding early cleavage in circulation. This linker based antibody drug conjugate (ADC) exhibited better antitumor activity.

## 5. Nanodevices

As mentioned above, there have been many varieties of polymers, dendrimers, and proteins used to conjugate with paclitaxel as carriers. Besides these carriers, nanodevices have also been employed in developing paclitaxel prodrugs. Yuan and co-workers [135] developed an approach to bond paclitaxel to fluorescent mesoporous silica nanoparticles (FMSN) covalently via disulfide linker which was sensitive to glutathione concentration. This conjugate could load up to 13% by weight. *In vitro* study showed this conjugate could be effectively taken up by Hela cells with reduced toxicity and side effects. Fluorescent silicon oxide based paclitaxel conjugate also showed great potential for clinical use [136].

In order to get better water solubility, Ding and co-workers [137] added PEG spacer to gold nanoparticles to form thiol terminated PEG-paclitaxel-conjugates. Water solubility was enhanced by 4.6 × 10^5^ times compared to free paclitaxel. The conjugate also exhibited improved cytotoxicity and prolonged circulation. Gibson and co-workers [138] first synthesized 2 nm gold nanoparticles conjugated with paclitaxel. In the study, paclitaxel was attached to a hexaethylene glycol linker at the 7-OH and then the linker was linked to phenol-terminated gold nanocrystals. This offered an opportunity to develop gold based conjugates.

Xu and co-workers [139] used PEGylated graphene oxide as drug carrier, paclitaxel was linked to PEG via a succinic liner. The resulting conjugate showed high water solubility and bioavailability, and could be quickly absorbed by lung cancer cell A549 and breast cancer MCF7. Besides graphene, fullerene could also be employed as drug carrier. Zakharian and co-workers [140] developed a fullerene-paclitaxel conjugate to realize slow-release property and also made a single dose “drug cocktail” for paclitaxel possible.

## 6. Conclusions

Paclitaxel is a promising antitumor agent which was originally separated from natural plants. However, there are some limitations towards its wide clinical application. Hence, efforts to obtain smarter targeting paclitaxel have never stopped. In the early stage of paclitaxel’s development strategies, studies mainly focused on the problem of low water solubility. As more and more research groups contributed to paclitaxel’s preparation, diverse prodrug techniques were used to obtain more efficient paclitaxel. With modern preparation methods, the anti-tumor activity of paclitaxel can be enhanced a great deal. However, for improved formulation or drug combination, there still remains a lot of work to be carried out by scientists in the future. Furthermore, recently many groups have published their works on self-assembly nano particles which can be prepared easily. This may be the trend to replace traditional complicated preparation manners and formulation. Besides drug formulation, enhancing the targeting ability of paclitaxel with fewer side effects is also a challenge. In the traditional way, paclitaxel prodrugs can achieve targeting ability by employing targeting moieties including tumor cell over-expressed proteins recognizing peptide or small molecules, and employing linkers which are sensitive to the tumor environment. Whether there is a better way for paclitaxel to obtain targeting activity is still under development.

## Figures and Tables

**Table 1:** cLogP and t_1/2_ of silicated paclitaxel prodrugs with different alkyl groups.

2′-O	7-O	cLogP	T_1/2_ (min)
H	H	3.2	N/A
Si(OEt)_3_	H	5.0	3.7
Si(O*n*-Oct)_3_	H	7.7	12
Si(O*i*-Pr)	H	5.6	120
Si(O*t*-Bu)_2_(OEt)	H	5.8	12,000
Si(Omenthyl)_3_	H	7.4	69,000
H	Si(OEt)_3_	5.1	30
H	Si(O*n*-Oct)_3_	7.8	150
Si(OEt)_3_	Si(OEt)_3_	6.3	4.6 (2′-O) 33 (7-O)
